# Community Health Worker Interventions Addressing Social Determinants of Health in Chronic Disease: A Systematic Review

**DOI:** 10.5888/pcd23.260132

**Published:** 2026-10-29

**Authors:** Marcee Wilder, Aleksandra Degtyar, Jasmine McKay, Shruti Mavuri, Mia Bolton, Lynne D. Richardson, Kimberly Souffront

**Affiliations:** 1 Department of Emergency Medicine, Icahn School of Medicine at Mount Sinai, New York, New York; 2 Institute for Health Equity Research, Icahn School of Medicine at Mount Sinai, New York, New York; 3 Department of Population Health Science and Policy, Icahn School of Medicine at Mount Sinai, New York, New York; 4 Howard University College of Medicine, Washington, District of Columbia; 5 Levy Library, Icahn School of Medicine at Mount Sinai, New York, New York; 6 Center for Nursing Research and Innovation, Mount Sinai Health System, New York, New York

## Abstract

**Introduction:**

Chronic diseases disproportionately affect people facing social and economic adversity. Community health workers (CHWs) are increasingly recognized as a key workforce for addressing social determinants of health (SDOH). However, evidence for the effect of CHW-led interventions on social and clinical outcomes among US adults with chronic disease is limited. This systematic review evaluated the effects of CHW-led interventions on SDOH and health outcomes.

**Methods:**

Following PRISMA guidelines and a registered Prospero protocol, we searched PubMed, Scopus, and Embase (Ovid) in April 2024 for US-based randomized or quasi-experimental studies involving adults with chronic disease and a patient-facing CHW intervention addressing at least 1 SDOH domain. Eligible studies were required to have a comparator group and report social or health outcomes. We used standardized forms to extract data and assess quality.

**Results:**

We identified 1,939 records and screened 1,058 abstracts. We reviewed 110 full-text articles; 7 studies met inclusion criteria. All were randomized controlled trials addressing at least 1 SDOH domain. Interventions most commonly targeted health care access, housing stability, or care coordination among adults with diabetes, HIV, asthma, hepatitis C, or hearing loss. CHW interventions demonstrated improvements in several condition-specific clinical outcomes, but overall effects on major clinical indicators were modest and inconsistent.

**Conclusion:**

CHW-led interventions show promise for addressing social needs and improving engagement in care among adults with chronic disease, but evidence of meaningful clinical benefit is still limited. The lack of large, well-powered trials underscores the need for more rigorous research to strengthen the evidence base for CHW-driven SDOH interventions in US health systems.

**Summary**

**What is already known on this topic?**

Community health workers (CHWs) are widely used to support chronic disease management and are increasingly promoted as a strategy to address social determinants of health (SDOH), yet evidence linking CHW-led SDOH interventions to measurable health outcomes is inconsistent.

**What is added by this report?**

This systematic review of US-based randomized controlled trials found that CHW-led interventions consistently improved engagement in care and addressed social needs but demonstrated only modest and variable effects on clinical outcomes.

**What are the implications for public health practice?**

Rigorous, adequately powered trials using standardized SDOH measures are needed to guide sustainable integration of CHWs into chronic disease programs.

## Introduction

The increasing prevalence of chronic disease in the US presents both a clinical and societal challenge, reflecting the environments in which people manage their health (1). More than half of US adults currently live with at least 1 chronic condition, with many experiencing multiple conditions, positioning chronic illness as a primary contributor to disability and premature mortality and escalating health care costs (1). Although effective treatments are available, their efficacy is frequently constrained by the social and economic factors that influence daily life (1). Social determinants of health (SDOH) refer to the conditions in which people are born, live, work, and age that influence health outcomes. Clinical care alone is insufficient to address the barriers encountered by people facing adverse SDOH, such as unstable housing, food insecurity, unreliable transportation, or financial hardship, all of which substantially affect health outcomes (1,2).

The World Health Organization emphasizes the critical influence of SDOH on the management of chronic disease (3). One approach increasingly used to address this intersection of medical care and social need is the integration of community health workers (CHWs). As trusted frontline public health professionals, the responsibilities of CHWs include facilitating access to social services, helping to navigate health insurance, securing food and housing support, managing medications, and providing culturally relevant education (4). CHWs are uniquely positioned to address SDOH due to their close ties to the communities they serve, their ability to build trust, and their role in bridging health care systems and social services (5). A growing body of literature shows that CHWs can improve chronic disease management, increase access to preventive care, enhance patient experiences, and contribute to reduced health care costs; however, this literature has largely focused on disease-specific outcomes, rather than the effect of CHWs on mitigation of social barriers (4). A comprehensive synthesis is needed to determine whether CHW-led interventions effectively address SDOH and whether these changes facilitate improvements in chronic disease management. The objective of this systematic review was to examine how CHW-led interventions address SDOH among adults with chronic disease and to summarize themes that emerged across studies. Although CHW programs have been implemented globally, including in low- and middle-income countries, where they have demonstrated effectiveness in improving health outcomes, the roles, training, and integration of CHWs in health care systems vary across settings (6). We, therefore, restricted this review to US-based studies to ensure relevance to domestic health care delivery models, workforce structures, and reimbursement environments.

## Methods

### Data sources

Our systematic review followed the Preferred Reporting Items for Systematic Reviews and Meta-Analyses (PRISMA) (7) guidelines, and we registered our protocol with Prospero, an international database of systematic reviews across health and social care disciplines (8). The objective of our search was to identify all studies that examined how CHWs influenced at least 1 SDOH outcome among patients with at least 1 chronic disease. For CHWs, search terms included commonly used synonymous roles, such as lay health workers and promotoras (Table 1). We used the Healthy People 2030 SDOH model (9) to define SDOH factors. This model comprises 5 domains: 1) economic stability, 2) education access and quality, 3) health care access and quality, 4) neighborhood and built environment, and 5) social and community context. With the help of a librarian (M.B.), the lead author (M.W.) identified keywords and relevant Medical Subject Headings (MeSH) terms that aligned with a Healthy People 2030 domain and a CHW intervention. We used broad MeSH terms and keywords to identify interventions addressing these domains, regardless of whether studies explicitly labeled them as SDOH. Once the search terms were defined, the librarian performed an electronic search in PubMed, Scopus, and Embase (Ovid) in April 2024 for potential studies for inclusion in the review. We did not conduct a supplementary search.

**Table 1:** Search Terms for Systematic Review of US Studies on Community Health Worker Interventions Addressing Social Determinants of Health in Chronic Disease^a^

Database	Community health workers	Chronic disease	Social determinants of health
PubMed (n = 529)	(“Community Health Workers”[Mesh] OR (“community health”[tiab] AND (agent[tiab] OR agents[tiab] OR volunteer[tiab] OR volunteers[tiab] OR worker[tiab] OR workers[tiab] OR attendant[tiab] OR attendants[tiab])))	(“Chronic Disease”[Mesh] OR “chronic disease*”[tiab] OR “chronic symptom*”[tiab] OR “Chronic Illness*”[tiab] OR “Chronically Ill”[tiab] OR “Chronic Condition*”[tiab] OR “chronic health condition*”[tiab] OR “Long-Term Illness*”[tiab] OR “long-term condition*”[tiab] OR “Persistent Disease*”[tiab] OR “persistent condition*”[tiab] OR “long lasting health condition”[tiab:~1] OR “chronic inflammation”[tiab] OR “chronic inflammatory”[tiab] OR “inflammatory chronic”[tiab] OR “chronic injury*”[tiab] OR “chronic wound*”[tiab] OR “chronic heart disease*”[tiab] OR “heart disease*”[tiab] OR “chronic coronary disease*”[tiab] OR “heart failure”[tiab] OR “coronary artery disease*”[tiab] OR “congenital heart disease*”[tiab] OR “valvular heart disease*”[tiab] OR “Heart Diseases”[Mesh] OR hypertension[tiab] OR “Hypertension”[Mesh] OR “high blood pressure”[tiab] OR hyperlipidemia[tiab] OR “Hyperlipidemias”[Mesh] OR hypercholesterolemia[tiab] OR “Hypercholesterolemia”[Mesh] OR hypertriglyceridemia[tiab] OR “Hypertriglyceridemia”[Mesh] OR “chronic obstructive pulmonary disease”[tiab] OR COPD[tiab] OR “Lung Diseases, Obstructive”[Mesh] OR “Asthma-Chronic Obstructive Pulmonary Disease Overlap Syndrome”[Mesh] OR “Pulmonary Disease, Chronic Obstructive”[Mesh] OR “pulmonary fibrosis”[tiab] OR “Pulmonary Fibrosis”[Mesh] OR “pulmonary disease”[tiab] OR “chronic obstructive lung”[tiab] OR “lung disease”[tiab] OR “Asthma”[Mesh] OR asthma[tiab] OR “diabetes”[tiab] OR “type 2 diabetes”[tiab] OR “type two diabetes”[tiab] OR “diabetes mellitus”[tiab] OR “Diabetes Mellitus”[Mesh] OR osteoporosis[tiab] OR “Osteoporosis”[Mesh] OR crohn’s[tiab] OR “Crohn Disease”[Mesh] OR “ulcerative colitis”[tiab] OR “Colitis, Ulcerative”[Mesh] OR “chronic inflammatory bowel disease”[tiab] OR “Inflammatory Bowel Diseases”[Mesh] OR “irritable bowel syndrome”[tiab] OR “Irritable Bowel Syndrome”[Mesh] OR “Alzheimer Disease”[Mesh] OR Alzheimer’s[tiab] OR Parkinson’s[tiab] OR “Parkinson Disease”[Mesh] OR “multiple sclerosis”[tiab] OR “Multiple Sclerosis”[Mesh] OR “amyotrophic lateral sclerosis”[tiab] OR “muscular dystrophy”[tiab] OR HIV[tiab] OR AIDS[tiab] OR “hepatitis B”[tiab] OR “hepatitis C”[tiab] OR “Arthritis, Rheumatoid”[Mesh] OR “rheumatoid arthritis”[tiab] OR fibromyalgia[tiab] OR osteoarthritis[tiab] OR “chronic kidney disease”[tiab] OR CKD[tiab] OR “chronic bladder”[tiab] OR “interstitial cystitis”[tiab] OR “urinary incontinence”[tiab] OR “Urinary Incontinence”[Mesh] OR hyperphosphatemia[tiab] OR “Hyperphosphatemia”[Mesh] OR “chronic renal failure”[tiab] OR endometriosis[tiab] OR “Endometriosis”[Mesh] OR “polycystic ovary syndrome”[tiab] OR “Polycystic Ovary Syndrome”[Mesh] OR PCOS[tiab] OR “pelvic inflammatory disease”[tiab:~1] OR “Pelvic Inflammatory Disease”[Mesh] OR “chronic reproductive disease”[tiab:~1] OR “secondary hyperthyroidism”[tiab] OR “chronic autoimmune condition”[tiab:~1] OR autoimmune[tiab] OR autoimmunity[tiab] OR “immune system disease”[tiab] OR “immune system diseases”[tiab] OR “Autoimmune Diseases”[Mesh])	((“Social Determinants of Health”[Mesh] OR “Healthcare Disparities”[Mesh] OR “health equity”[MeSH] OR “social isolation”[Mesh] OR “social determinant of health”[tiab] OR “health equity”[tiab] OR “health equities”[tiab] OR “health inequity”[tiab] OR “health inequities”[tiab] OR “social health”[tiab] OR “social care”[tiab] OR “social medicine”[tiab] OR “contextualized care”[tiab] OR “contextual care”[tiab] OR “social risk”[tiab] OR “social need”[tiab] OR “social context”[tiab] OR “social determinants”[tiab] OR “Structural Determinants”[tiab] OR “structural competency”[tiab] OR “structural competence”[tiab] OR “Community-oriented primary care”[tiab] OR “health disparities”[tiab] OR “health disparity”[tiab] OR “health equity”[tiab] OR “whole person care”[tiab] OR “Financial security”[tiab] OR “financial insecurity”[tiab] OR “Transportation”[tiab] OR “Food security”[tiab] OR “Food insecurity”[tiab] OR “housing security”[tiab] OR “housing insecurity”[tiab] OR “Hunger”[tiab] OR “Homeless”[tiab] OR “Homelessness”[tiab] OR “Housing”[tiab] OR “social isolation”[tiab] OR “poverty”[tiab] OR “social prescribing”[tiab] OR “community referral”[tiab]))
Scopus (n = 621)	TITLE-ABS-KEY({community health agent} OR {community health agents} OR {community health volunteer} OR {community health volunteers} OR {community health worker} OR {community health workers} OR {community health attendant} OR {community health attendants})	TITLE-ABS-KEY({chronic disease} OR {chronic symptom} OR {chronic illness} OR {chronically ill} OR {chronic condition} OR {chronic health condition} OR {long-term illness} OR {long-term condition} OR {persistent disease} OR {persistent condition} OR {long lasting health condition} OR {chronic inflammation} OR {chronic inflammatory} OR {inflammatory chronic} OR {chronic injury} OR {chronic wound} OR {chronic heart disease} OR {heart disease} OR {chronic coronary disease} OR {heart failure} OR {coronary artery disease} OR {congenital heart disease} OR {valvular heart disease} OR {hypertension} OR {high blood pressure} OR {hyperlipidemia} OR {hypercholesterolemia} OR {hypertriglyceridemia} OR {chronic obstructive pulmonary disease} OR {COPD} OR {pulmonary fibrosis} OR {pulmonary disease} OR {chronic obstructive lung} OR {lung disease} OR {asthma} OR {diabetes} OR {type 2 diabetes} OR {type two diabetes} OR {diabetes mellitus} OR {osteoporosis} OR {crohn} OR {ulcerative colitis} OR {chronic inflammatory bowel disease} OR {irritable bowel syndrome} OR {Alzheimer} OR {Parkinson} OR {multiple sclerosis} OR {amyotrophic lateral sclerosis} OR {muscular dystrophy} OR {HIV} OR {AIDS} OR {hepatitis B} OR {hepatitis C} OR {rheumatoid arthritis} OR {fibromyalgia} OR {osteoarthritis} OR {chronic kidney disease} OR {CKD} OR {chronic bladder} OR {interstitial cystitis} OR {urinary incontinence} OR {hyperphosphatemia} OR {chronic renal failure} OR {endometriosis} OR {polycystic ovary syndrome} OR {PCOS} OR {pelvic inflammatory disease} OR {chronic reproductive disease} OR {secondary hyperthyroidism} OR {chronic autoimmune condition} OR {autoimmune} OR {autoimmunity} OR {immune system disease} OR {immune system diseases})	TITLE-ABS-KEY(({social determinant of health} OR {health equity} OR {health equities} OR {health inequity} OR {health inequities} OR {social health} OR {social care} OR {social medicine} OR {contextualized care} OR {contextual care} OR {social risk} OR {social need} OR {social context} OR {social determinants} OR {structural determinants} OR {structural competency} OR {structural competence} OR {community-oriented primary care} OR {health disparities} OR {health disparity} OR {health equity} OR {whole person care} OR {financial security} OR {financial insecurity} OR {transportation} OR {food security} OR {food insecurity} OR {housing security} OR {housing insecurity} OR {hunger} OR {homeless} OR {homelessness} OR {housing} OR {social isolation} OR {poverty} OR {social prescribing} OR {community referral}))
Embase (Ovid) (n = 789)	(‘community health worker’.mp. or ‘community health workers’.mp. or ‘community health agent’.mp. or ‘community health agents’.mp. or ‘community health volunteer’.mp. or ‘community health volunteers’.mp. or ‘community health attendant’.mp. or ‘community health attendants’.mp. OR exp health auxiliary/)	(chronic disease.mp. or exp chronic disease/ or ‘chronic disease*’.mp. or ‘chronic symptom*’.mp. or ‘Chronic Illness*’.mp. or ‘Chronically Ill’.mp. or ‘chronic condition*’.mp. or ‘chronic health condition*’.mp. or ‘long-term illness*’.mp. or ‘long-term condition*’.mp. or ‘persistent disease*’.mp. or ‘persistent condition*’.mp. or ‘long condition’.mp. or ‘chronic inflammation’.mp. or ‘chronic inflammatory’.mp. or ‘inflammatory chronic’.mp. or ‘chronic injur*’.mp. or ‘chronic wound*’.mp. or ‘chronic heart disease*’.mp. or ‘heart disease*’.mp. or ‘chronic coronary disease*’.mp. or ‘heart failure’.mp. or ‘coronary artery disease*’.mp. or ‘congenital heart disease*’.mp. or ‘valvular heart disease*’.mp. or ‘hypertension’.mp. or ‘high blood pressure’.mp. or ‘hyperlipidemia’.mp. or ‘hypercholesterolemia’.mp. or ‘hypertriglyceridemia’.mp. or ‘chronic obstructive pulmonary disease’.mp. or ‘COPD’.mp. or ‘pulmonary fibrosis’.mp. or ‘pulmonary disease’.mp. or ‘chronic obstructive lung’.mp. or ‘lung disease’.mp. or ‘asthma’.mp. or ‘diabetes’.mp. or ‘type 2 diabetes’.mp. or ‘type two diabetes’.mp. or ‘diabetes mellitus’.mp. or ‘osteoporosis’.mp. or ‘crohn’.mp. or ‘ulcerative colitis’.mp. or ‘chronic inflammatory bowel disease’.mp. or ‘irritable bowel syndrome’.mp. or ‘alzheimer’.mp. or ‘parkinson’.mp. or ‘multiple sclerosis’.mp. or ‘amyotrophic lateral sclerosis’.mp. or ‘muscular dystrophy’.mp. or ‘HIV’.mp. or ‘AIDS’.mp. or ‘hepatitis B’.mp. or ‘hepatitis C’.mp. or ‘rheumatoid arthritis’.mp. or ‘fibromyalgia’.mp. or ‘osteoarthritis’.mp. or ‘chronic kidney disease’.mp. or ‘CKD’.mp. or ‘chronic bladder’.mp. or ‘interstitial cystitis’.mp. or ‘urinary incontinence’.mp. or ‘hyperphosphatemia’.mp. or ‘chronic renal failure’.mp. or ‘endometriosis’.mp. or ‘polycystic ovary syndrome’.mp. or ‘PCOS’.mp. or ‘pelvic disease’.mp. or ‘secondary hyperthyroidism’.mp. or ‘autoimmune’.mp. or ‘autoimmunity’.mp. or ‘immune system disease’.mp. or ‘immune system diseases’.mp.)	(‘social determinants of health’.mp. or exp social determinants of health/ or ‘health equity’.mp. or ‘health equities’.mp. or ‘health inequity’.mp. or ‘health inequities’.mp. or ‘social health’.mp. or ‘social care’.mp. or ‘social medicine’.mp. or ‘contextualized care’.mp. or ‘contextual care’.mp. or ‘social risk’.mp. or ‘social need’.mp. or ‘social context’.mp. or ‘social determinants’.mp. or ‘structural determinants’.mp. or ‘structural competency’.mp. or ‘structural competence’.mp. or ‘community-oriented primary care’.mp. or ‘health disparities’.mp. or ‘health disparity’.mp. or ‘whole person care’.mp. or ‘financial security’.mp. or ‘financial insecurity’.mp. or ‘transportation’.mp. or ‘food security’.mp. or ‘food insecurity’.mp. or ‘housing security’.mp. or ‘housing insecurity’.mp. or ‘hunger’.mp. or ‘homeless’.mp. or ‘homelessness’. MP. or ‘housing’.mp. or ‘social isolation’.mp. or ‘poverty’.mp. or ‘social prescribing’.mp. or ‘community referral’.mp.)

a The search was conducted in April 2024. Eligibility requirements included being published in English in a peer-reviewed journal in 2000 or later.

### Study selection

We applied predefined inclusion and exclusion criteria to determine study eligibility. Studies were included if they 1) were original research conducted in the US, 2) enrolled adult participants aged 18 years or older with a chronic disease, defined by the Centers for Disease Control and Prevention as “conditions that last one year or more and require ongoing medical attention or limit activities of daily living or both” (2), 3) evaluated a CHW-led, patient-facing intervention addressing at least 1 SDOH domain, 4) used a prospective or retrospective design and randomized controlled or quasi-experimental methods with a comparison group, 5) reported measurable outcomes related to SDOH improvement, chronic disease outcomes, or health care utilization, and 6) were published in English in peer-reviewed journals in 2000 or later. We defined CHW-led interventions as those in which CHWs were the people primarily responsible for delivering patient-facing components of the intervention, including education, care coordination, or navigation to health and social services, even when embedded within multidisciplinary teams. We limited inclusion to studies published in 2000 or later to reflect contemporary health care delivery models and the evolving integration of CHWs within modern US health systems (10).

We excluded studies that 1) were published before 2000 or conducted outside the US, 2) were systematic reviews, commentaries, or opinion pieces, 3) were observational or cohort studies without an intervention or comparison group, 4) evaluated feasibility or acceptability only, or 5) did not include a CHW-led or patient-facing intervention addressing an SDOH domain.

We imported all references identified through the database search into Covidence, an online systematic review management platform (11). Covidence automatically detected and removed duplicate records before screening. All references were screened independently by at least 2 reviewers at both the title and abstract and full-text stages using Covidence. The entire team resolved conflicts through discussion and consensus.

### Data extraction

Two reviewers (M.W., K.S.) independently extracted data using a standardized extraction form that captured information on study characteristics, participant populations, intervention components, outcomes, and results. Discrepancies were resolved through discussion and consensus. Findings were synthesized descriptively because of heterogeneity in interventions and outcomes.

### Data synthesis and analysis

After data extraction, we conducted an iterative descriptive analysis of the included studies rather than a meta-analysis. We distinguished between “addressing” SDOH, defined as identifying needs and facilitating referrals or connections to resources, and “resolving” SDOH, defined as documented improvement or elimination of a social barrier. Included studies inconsistently defined and measured these constructs. All included studies were randomized controlled trials (RCTs) that examined CHW-led interventions addressing SDOH; however, differences in intervention design, outcomes, and chronic disease populations supported a qualitative synthesis. We analyzed each included study to descriptively and qualitatively summarize study and intervention characteristics and identify emergent themes. We used the Risk of Bias 2 tool (12) to assess risk of bias for each included study. The ROBINS–I (Risk of Bias in Non-randomized Studies – of Interventions) tool (13) had been prespecified for eligible nonrandomized quasi-experimental studies, but no such studies met inclusion criteria. The overall synthesis, therefore, focused on comparing and contrasting the included studies and highlighting trends and gaps.

## Results

### Study selection

We identified 1,939 records for screening. After removing 881 duplicates, we screened 1,058 titles and abstracts, resulting in 110 full-text articles assessed for eligibility. Of these, we initially excluded 102, thus yielding 8 studies that initially met inclusion criteria; however, we excluded another study after full-text consensus review because the intervention did not meet criteria for a CHW-led, patient-facing intervention, thus yielding 7 included studies (Figure).

**Figure:**
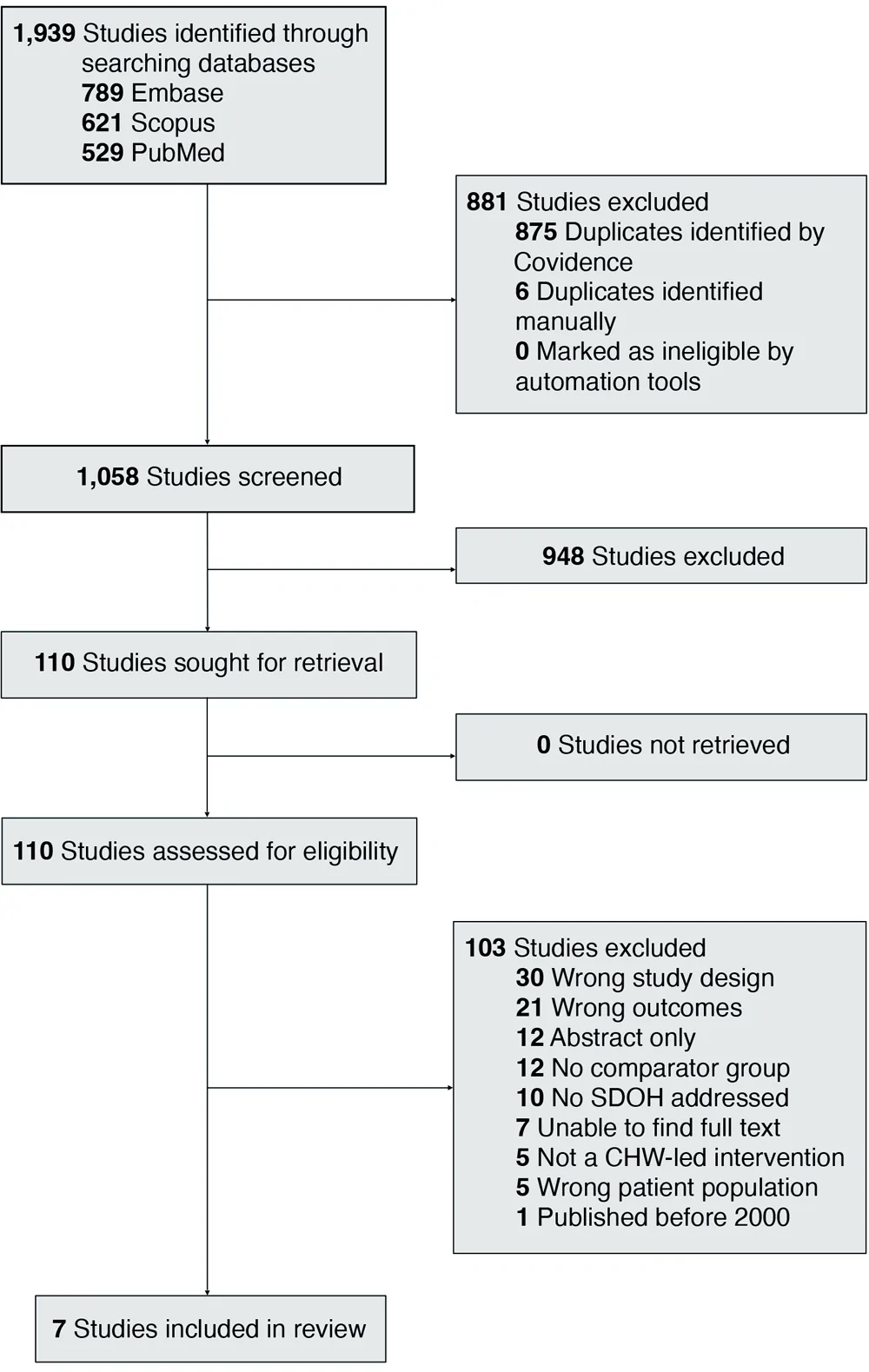
Flowchart for selection of studies in a systematic review of community health worker (CHW) interventions addressing social determinants of health (SDOH) in chronic disease.

### Study and intervention characteristics

All 7 studies were RCTs (Table 2): 3 were pilot RCTs and 4 were full-scale RCTs, enrolling small to modest numbers of participants (range, 10–366). All studies were primarily conducted in urban settings: Baltimore, Maryland (14); Portland, Oregon, within Kaiser Permanente Northwest (15); Dallas, Texas (16); Seattle–King County, Washington (17,18); Chicago, Illinois (19); and Los Angeles, California (20). CHWs were trained to deliver multicomponent interventions encompassing education (14,15,17–19), navigation and linkage to care (15,16,20), and case management or care coordination (16,18,19) to address clinical management and SDOH, including housing stability (20), access to health care (15,16,18), resource utilization (17,19), and social connectedness and quality of life (14).

**Table 2:** Summary of Included Studies and Outcomes in Systematic Review of US Studies on CHW Interventions Addressing Social Determinants of Health in Chronic Disease^a^

Characteristic	Nieman et al (14), 2022	Fitzpatrick et al (15), 2022	Hoff et al (16), 2023	Krieger et al (17), 2015	Nelson et al (18), 2017	Sharp et al (19), 2018	Nyamathi et al (20), 2023
**Study design**	RCT	Pilot RCT	Pilot RCT	RCT	RCT	RCT	Exploratory Pilot RCT
**Setting and population**	Affordable housing, senior centers, social clubs (Baltimore, Maryland)	Kaiser Permanente Northwest; CHWs embedded in Portland, Oregon, community-based organizations	County jail; recently incarcerated adults (Dallas, Texas)	Community-based (King County, Washington)	Community setting (Seattle–King County, Washington)	Primary care clinics (Chicago, Illinois)	Skid Row; adults experiencing homelessness (Los Angeles, California)
**Sample size (intervention vs control), no.**	78 vs 73	56 vs 54	17 vs 14	177 vs 189	145 vs 142	120 vs 124	4 vs 6
**Study duration/follow-up**	3-month randomized endpoint; 12-month postintervention follow-up	6 months	6 months (primary endpoint); follow-up through 12 months	12 months	12 months	12- and 24-month follow-up	6 months
**CHW role and intervention components**	CHW-facilitated delivery of over-the-counter hearing technology; education on device use	CHW needs assessment; diabetes self-management education	CHW navigation and needs assessment; service linkage	CHW education; asthma management planning; social service referrals; provision of home asthma supplies	CHW home visits; goal-setting; diabetes care planning	CHW home visits; accompaniment to primary care physician/pharmacy visits; diabetes education; social support; referral to resources	CHW education, barrier assessment, substance use referral, housing navigation
**Comparison group**	Wait-list control	Patient navigation to medical and community services	Usual care	Usual asthma care and educational materials	Usual care	Pharmacist-led medication management and health promotion	Clinic-based usual care
**Chronic condition**	Age related hearing loss	Diabetes	HIV	Asthma	Diabetes	Diabetes	Hepatitis C; substance use disorder
**SDOH domains addressed**	Low income; social participation; quality of life	Low income; food access; housing; transportation; utilities; insurance	Housing stability; food insecurity; employment; health care utilization	Low income; housing conditions; access to resources	Low income; health care utilization; health-related quality of life	Low income; quality of life; access to care	Housing instability; access to care; mental health
**Clinical outcomes**	Improved self-perceived communication function	Clinically significant HbA_1c_ reduction in both groups	No significant change in viral suppression	Clinically significant improvement in symptom-free days	No significant change in HbA_1c_	No significant change in HbA_1c_	Sustained virologic response among participants completing treatment
**Behavioral or process outcomes**	Improved use of hearing support	Improved engagement with social services	No significant change in medication adherence or health care utilization	Improved asthma self-management	15% fewer self-reported physician visits (*P* < .001); no significant difference in ED visits	Improved care coordination	Improved access to mental and physical health services
**SDOH outcomes**	Significant improvement in physical health–related quality of life	Greater proportion of social needs addressed in intervention group	Improved food security, housing stability, and employment	Significant improvement in asthma-related quality of life	No significant change in physical or mental quality of life	No significant change in perceived social support	Improved service access
**Psychosocial outcomes**	Improved listening self-efficacy; no significant between-group differences in depression, loneliness, social isolation, valuation of life, or technology-related self-efficacy	Qualitative interviews identified increased perceived control over diabetes management among some participants supported by patient navigators and a diabetes self-management support program facilitated by CHWs	Not reported	Improved physical health status; no significant between-group difference in mental health	No significant between-group differences in diabetes-related social burden	No significant between-group differences in quality of life	No significant between-group difference in mental health

Abbreviations: CHW, community health worker; ED, emergency department; HbA_1c_, glycated hemoglobin A_1c_; RCT, randomized controlled trial; SDOH, social determinants of health.

a The search was conducted in April 2024. Eligibility requirements included being published in English in a peer-reviewed journal in 2000 or later.

Descriptions of CHWs varied across studies; most CHWs were trained to provide education, navigation, and care coordination (14–20); 3 studies explicitly reported CHW lived experience or community concordance, such as use of formerly incarcerated CHWs (16) or CHWs recruited from the communities they served (17,18). CHWs received structured training and supervision across diverse models, including nurse-led (20), physician-supervised (18), and multidisciplinary teams involving pharmacists (19), clinicians (15), and researchers (18). Although models varied, several interventions demonstrated interprofessional collaboration, particularly within nurse–CHW (20) and CHW–pharmacist partnerships (19), which emphasized bidirectional communication (19), care coordination (18,20), and continuity across clinical and community settings. No study described continuity of care in the emergency department setting.

### Quality ratings

Overall study quality ranged from low (14,17) to moderate (15,16,18–20); no studies were rated as having high risk of bias across all domains. RCTs demonstrated moderate methodological rigor; however, several were limited by small sample sizes, particularly a study conducted in a county jail (n = 31 participants) (16) and a study conducted among adults experiencing homelessness (n = 10 participants) (20). Follow-up durations varied across studies and were generally short to moderate, with primary randomized follow-up conducted at 3 months (14), 6 months (15,16,20), or 12 months (17–19); Sharp et al additionally reported 24-month outcomes (19).

### Participant characteristics

Across the 7 studies, mean (SD) age of participants was 52.5 (23.5) years, with study-level means ranging from 35.9 to 76.7 years (14). Participants were predominantly from racial and ethnic minority populations, with Black adults comprising 16.7% (17) to 73.4% (19) and Hispanic adults 3% (15) to 46.7% (17) of study populations across studies. Sex distribution was relatively balanced overall (female 26.0 %–73.2%; male 26.8%–74.0 %). However, the study addressing incarceration (24 of 31 were male; 77.4 %) (16) and the study addressing homelessness (8 of 10 were male; 80.0%) (20) had a higher proportion of male participants than other studies. Participants had at least 1 chronic or high-risk condition, including HIV (16), diabetes (15,18,19), hepatitis C (20), age-related hearing loss (14), or asthma (17). All studies focused on people experiencing social or structural disadvantages, such as homelessness (20), incarceration (16), or low income (14,15,18,19).

### CHW effect on measurable outcomes

#### Clinical outcomes

We found significant improvement in select clinical outcomes, including asthma control (an increase of 2.02 [95% CI, 0.94–3.09] symptom-free days; *P* < .001) (17), self-perceived communication function, with an estimated treatment effect of −12.98 points (95% CI, −15.51 to −10.42) in the Hearing Handicap Inventory for the Elderly–Screening Version (HHIE-S; range, 0–40, with higher scores indicating poorer function) (14), and engagement in hepatitis C virus (HCV) treatment among people experiencing homelessness (20). In this small pilot study on HCV treatment (20), 3 of 4 intervention participants and 4 of 6 control participants achieved the intent-to-treat outcome of sustained virologic response at 12 weeks (SVR12) (*P* = .67); all 7 participants who completed SVR12 testing had undetectable HCV RNA. No significant improvements were observed for glycemic control among participants with diabetes in any of the studies focused on diabetes (15,18,19). Lastly, Hoff et al found no significant difference in HIV viral suppression at 6 months between CHW+ and treatment-as-usual participants (20% vs 37%, respectively; *P* = .61) (16).

#### Behavioral or process outcomes

Our review found improvements in diabetes self-management confidence. In qualitative interviews, participants receiving navigation-plus-CHW–delivered diabetes self-management support described improvements in eating habits, physical activity, blood glucose management, medication adherence, and perceived control over diabetes management (15). Nelson et al reported 15% fewer self-reported physician visits in the intervention group (*P* < .001) (18). Sharp et al found no between-group difference in self-reported medication adherence, although the estimated number of lipid-lowering medication intensification events was greater with pharmacist-plus-CHW support than with pharmacist support alone (0.39 [95% CI, 0.27–0.52] vs 0.26 [95% CI, 0.14–0.38]; *P* < .001) (19). In Nyamathi et al, 3 of 4 intervention participants and 4 of 6 control participants completed the follow-up assessment at 5 or 6 months (20).

#### Psychosocial outcomes

Six studies assessed psychosocial outcomes; the remaining study (16) focused exclusively on clinical, behavioral, and social needs outcomes. CHW-led interventions demonstrated significant improvements in psychosocial outcomes, including asthma-related quality of life (between-group improvement was 0.50 [95% CI, 0.28–0.71] points; *P* < .001) (17) and physical health–related quality of life measured by the 12-Item Short Form Health Survey Physical Component Summary (range, 0–100; higher scores indicate better health), with an estimated treatment effect of 4.80 (95% CI, 1.09–8.36) points (14). However, no significant improvements were observed in health-related quality of life or mental health outcomes in diabetes-focused trials (18,19), or mental health–related quality of life. Nieman et al found no significant between-group differences in 3-month changes in mental health–related quality of life, depression, social network diversity, or loneliness (14).

#### SDOH outcomes

Not all studies addressed the same SDOH domains; domains varied based on the included participants and intervention design. Although all 7 studies were conducted among populations experiencing social and structural disadvantages and incorporated intervention components designed to address SDOH, only 3 studies explicitly measured changes in SDOH outcomes (15,16,20). Studies often reported that social needs were “addressed” (eg, through referral or service linkage), but they did not consistently demonstrate resolution of those needs. Nyamathi et al reported significantly greater access to physical health services in the registered nurse (RN)–CHW intervention group compared with clinic-based standard care (20). Three of 3 participants in the RN–CHW intervention versus 0 of 5 participants in a clinic-based standard-care intervention reported access to physical health care at 5 or 6 months (*P* = .02), and 2 of 3 participants in the RN–CHW intervention versus 0 of 5 participants in the clinic-based standard-care intervention reported access to mental health services (*P* = .11) (20). In the study involving people recently released from incarceration (16), Hoff et al observed improvements in multiple economic and social stability indicators in the CHW intervention arm compared with the control. Among participants with SDOH follow-up data (10 CHW+ and 8 control participants), the proportion with stable housing increased by 52.4 percentage points in the CHW+ group and decreased by 7.2 points in the control group; full-time employment increased by 20.0 versus 4.6 percentage points, and food insecurity decreased by 32.5 versus 10.7 percentage points, respectively. Between-group statistical tests were not reported for these outcomes.

In a diabetes-focused pilot trial, Fitzpatrick et al found that participants receiving CHW support had a greater proportion of identified social needs addressed — including food access, housing, transportation, and utilities — compared with the control group, though effect sizes and *P* values for individual SDOH domains were not reported (15). In a large community-based trial involving people with asthma, Krieger et al operationalized neighborhood and built environment determinants through home-based assessments and referrals; although the study documented improvements in housing-related triggers and access to community resources, it did not quantify changes in SDOH as discrete outcomes (17).

## Discussion

This systematic review examined CHW-led interventions addressing SDOH among adults with chronic disease. Only 7 studies met inclusion criteria from more than 1,000 screened records, reflecting a limited and heterogeneous evidence base composed largely of full-scale RCTs conducted among populations at high risk of negative SDOH. Across the 7 RCTs, CHW-led interventions demonstrated a consistent pattern of stronger effects on engagement and social needs than on clinical disease indicators, which demonstrates the varied pathways through which CHWs may influence health outcomes.

CHW interventions produced modest and inconsistent improvements in clinical outcomes across the chronic disease conditions studied. The absence of consistent between-group differences in major biomarkers demonstrates the challenge of translating social and behavioral support into short-term physiologic change (15,16,18,19). Some condition-specific improvements were observed, such as better asthma symptom control and improved functional hearing; however, these tended to be isolated findings rather than consistent trends (14,17). Overall, the clinical effect of CHWs in these trials was limited, mirroring prior research demonstrating small improvements in physiologic outcomes (21). A meta-analysis of CHW-led programs for diabetes, for instance, found average reductions in HbA_1c_ of less than 1%, which aligns with the negligible between-group differences seen in our review (21). One trial showed that, among participants with very poorly controlled diabetes (baseline HbA_1c_ >10%), a decrease in HbA_1c_ was more than 1% larger in the group with CHW support than in the group with usual care (21). However, such subgroup findings require confirmation in larger, adequately powered trials. Overall, the lack of sustained clinical improvement likely reflects short follow-up periods, limited intervention intensity, and the complex pathways linking social support to biological outcomes.

In contrast to the generally modest effects on clinical outcomes, several studies demonstrated favorable behavioral or process outcomes, although findings were not uniformly significant. CHW support was associated with better medication adherence, higher rates of follow-up completion, and greater self-management confidence (15,16,20). These improvements were especially pronounced in studies tailored toward traditionally hard-to-reach populations, such as people experiencing homelessness or recent incarceration. Such findings illustrate how CHWs can build trust and provide navigation so that populations experiencing social and structural disadvantages remain connected to care. Improvements in intermediate outcomes, such as adherence, self-efficacy, and care coordination, are consistent with the well-documented strengths of CHW programs. Kim et al similarly found that CHWs often succeed in increasing use of preventive care and promoting chronic disease self-care among populations vulnerable to poor health outcomes (4). Sharp et al and Nelson et al both observed that CHWs facilitated more timely clinical decision-making, such as medication titration, by supporting patient follow-through and communication and feedback with care teams (18,19). Although these improvements did not consistently translate into measurable clinical improvements within study time frames, they represent important intermediate outcomes that are necessary for long-term disease control.

Psychosocial outcomes demonstrated mixed results. CHW-led interventions demonstrated improved disease-specific quality-of-life measures, such as asthma-related functioning (17) and hearing-related communication (14) but did not consistently improve broader measures of mental health or social isolation (18,19). These findings suggest that although CHWs may improve patients’ day-to-day functioning and disease-specific well-being (likely through providing social support, education, and empowerment), they may still fall short of addressing deeper or more complex psychosocial issues within the scope of a single intervention. Outcomes, such as depression or social isolation, may be influenced by many factors and require longer-term or specialized interventions beyond what a typical CHW program can offer in a short trial. These findings align with prior systematic evidence. In their comprehensive review of 67 RCTs, Kim et al found that CHW interventions were associated with improvements in disease-specific self-management behaviors, use of preventive care, and select cardiometabolic outcomes, including blood pressure and glycemic control, particularly among populations experiencing social and structural disadvantages. However, that review did not demonstrate consistent or robust effects on broader mental health or psychosocial outcomes, and measurement of social support and quality-of-life indicators was often heterogeneous or underreported (4). In the context of chronic disease care, CHWs appear to address some stressors and improve patients’ outlook on managing illness, but transforming overall psychosocial health remains challenging.

Although all included studies were designed for socially disadvantaged populations, only 3 explicitly measured patient-level SDOH outcomes (15,16,20) and 1 study operationalized social determinants through housing and built environment interventions (17). Although SDOH outcomes were inconsistently measured, the direction of effect across trials suggests that CHWs may be particularly effective in addressing immediate material barriers (eg, facilitating access to services), although few studies demonstrated resolution of these barriers. However, the absence of standardized SDOH metrics limits comparability and undermines confidence in pooled inference (15–17,20)

The scope of SDOH addressed by CHW interventions was relatively narrow and often tailored to the study population or setting rather than comprehensively addressing the full range of health-related social needs. Common domains included health care access and utilization, housing stability, food insecurity, and, to a lesser extent, employment and income assistance. No single intervention comprehensively addressed all social needs, which is unsurprising given resource constraints and the complexity of individuals’ circumstances. CHWs typically focused on immediate, actionable barriers to health management; although pragmatic, this approach may leave other unaddressed determinants in place (22). For example, securing stable housing without addressing food insecurity or unemployment may limit downstream health gains. These findings suggest that more comprehensive, multicomponent interventions or cross-sector partnerships may be necessary to fully address the interconnected SDOH affecting chronic disease outcomes.

A striking finding of this review is the lack of large, high-quality studies. Despite growing national enthusiasm for leveraging CHWs to address SDOH, we found only a handful of trials, 3 of which were pilot studies with few participants (range, 10–110), and others had substantial methodological limitations. We found that few trials were powered to detect clinically meaningful differences in health outcomes. We did not find any quasi-experimental evaluations with a comparator group of community interventions at the neighborhood or health system level, or any randomized implementation trials, suggesting a gap between community programs and published evaluation research. Across studies, risk of bias was most commonly rated as “some concerns,” driven by open-label designs, variable intervention fidelity, limitations in allocation concealment, and incomplete reporting of randomization (14–20). Several trials were further limited by missing outcome data or reliance on self-reported social or behavioral measures, increasing susceptibility to attrition and measurement bias (14,16,19,20). Although objective clinical outcomes were generally assessed using appropriate methods, inconsistent adherence and limited use of analytic approaches to address nonadherence or missingness constrained confidence in observed effect estimates (15,17–19).

The paucity of rigorous evidence poses challenges for translating interventions into policy and practice. Policymakers and health systems increasingly recognize CHWs as vital for addressing health-related social needs and recognize CHW interventions as a way to address health equity (4). And yet, evidence of the effect of CHWs on SDOH to support large investments or standardized models is weak.

To fully realize the potential of CHWs within health systems, future research must move beyond small pilot trials toward larger, methodologically rigorous studies that explicitly measure SDOH alongside clinical outcomes. Advancing this field will require standardized, validated SDOH measures to enable cross-study comparison, distinguish between addressing and resolving social needs, and strengthen inference for the effect of CHW-led interventions. Without such evidence, widespread implementation risks will outpace the science needed to guide sustainable policy and reimbursement models.

### Limitations

This review has several limitations. First, the underlying evidence base is limited. Most of our included studies have small samples or short follow-up periods, which constrained the ability to detect meaningful changes in health outcomes. Primary randomized follow-up ranged from 3 to 12 months, with 1 study reporting additional 24-month outcomes, potentially limiting the observation of sustained clinical or social improvements. Several studies also lacked strict blinding or clearly defined usual care conditions, introducing potential bias. Second, we found substantial heterogeneity in CHW training and supervision, targeted SDOH domains, and chronic disease type across studies. Chronic disease type and related outcomes varied widely, from asthma symptom–free days to HIV viral suppression to HbA_1C_ and patient-reported measures, which precluded our ability to perform a pooled quantitative analysis. Third, reporting of implementation fidelity was inconsistent: some studies described detailed CHW protocols, and others offered minimal information, thus limiting our ability to determine whether interventions labeled “CHW-led” were comparable across settings. Fourth, our review process had limitations. Restricting studies to the English language and US-based comparator trials may have excluded relevant studies. Fifth, we also excluded studies published before 2000, which may have omitted formative work. Sixth, publication bias is also possible, although the number of null primary outcomes reduces this concern. Seventh, our broad definition of SDOH may have introduced subjectivity, as interventions ranged from targeted supports to broader navigation efforts. Finally, SDOH outcomes were measured inconsistently, mostly through self-report rather than standardized instruments, with a few notable exceptions (16,20). Despite these limitations, we used rigorous systematic methods, including dual review and structured quality assessment. The small, heterogeneous evidence base does require cautious interpretation, but the trends identified provide direction for future research.

### Conclusion

This review demonstrates that CHW-led interventions show the most consistent effects on behavioral and engagement-related outcomes, including medication adherence, care continuity, and self-management confidence, among adults with chronic disease, yet the current evidence base is limited by small sample sizes, short follow-up periods, and heterogeneous measurement of SDOH outcomes. Improvements in psychosocial and disease-specific quality-of-life measures were mixed, and clinical outcomes were modest and inconsistent across trials. To move beyond promising pilot data, future trials must incorporate standardized SDOH metrics, longer follow-up, and adequately powered designs capable of detecting both social and clinical effects. Without such rigor, CHW programs risk being widely implemented without a commensurate evidence base to guide scale and sustainability.
